# Diagnostic and prognostic value of the HFA-PEFF score for heart failure with preserved ejection fraction: a systematic review and meta-analysis

**DOI:** 10.3389/fcvm.2024.1389813

**Published:** 2024-07-12

**Authors:** Xinmei Li, Yunyu Liang, Xiaozhong Lin

**Affiliations:** Department of Geriatrics, The Second Clinical College of Guangzhou University of Chinese Medicine, Guangdong Provincial Hospital of Chinese Medicine, Guangzhou, Guangdong, China

**Keywords:** HFA-PEFF, HFpEF, prognosis, diagnosis, meta-analysis

## Abstract

**Aim:**

To assess the diagnostic and prognostic performances of the Heart Failure Association Pre-test Assessment, Echocardiography & Natriuretic Peptide, Functional Testing, Final Etiology (HFA-PEFF) score for heart failure with preserved ejection fraction (HFpEF) in a comprehensive manner.

**Methods:**

PubMed, Embase, Cochrane Library, and Web of Science were comprehensively searched from the inception to June 12, 2023. Studies using the “Rule-out” or “Rule-in” approach for diagnosis analysis or studies on cardiovascular events and all-cause death for prognosis analysis were included. The Quality Assessment of Diagnostic Accuracy Studies (QUADAS−2) tool was adopted to assess the quality of diagnostic accuracy studies. The sensitivity (SEN), specificity (SPE), positive likelihood ratio (PLR), negative likelihood ratio (NLR), diagnostic odds ratio (DOR), and area under the summary receiver operating characteristic (SROC) curve (AUC) were presented with 95% confidence intervals (CIs). For CVEs and all-cause death, the hazard ratio (HR) values were calculated.

**Results:**

Fifteen studies involving 6420 subjects were included, with 9 for diagnosis analysis, and 7 for prognosis analysis. For the diagnostic performance of the HFA-PEFF score, with the “Rule-out” approach, the pooled SEN was 0.96 (95%CI: 0.94, 0.97), the pooled SPE was 0.39 (95%CI: 0.37, 0.42), and the pooled AUC was 0.85 (95%CI: 0.67, 1.00), and with the “Rule-in” approach, the pooled SEN was 0.59 (95%CI: 0.56, 0.61), the pooled SPE was 0.86 (95%CI: 0.84, 0.88), and the pooled AUC was 0.83 (95%CI: 0.79, 0.87). For the predictive performance of the HFA-PEFF score, regarding CVEs, the pooled SEN was 0.63 (95%CI: 0.58, 0.67), the pooled SPE was 0.53 (95%CI: 0.49, 0.58), and the pooled AUC was 0.65 (95%CI: 0.40, 0.90), and concerning All-cause death, the pooled SEN was 0.85 (95%CI: 0.81, 0.88), the pooled SPE was 0.48 (95%CI: 0.44, 0.52), and the pooled AUC was 0.65 (95%CI: 0.47, 0.83). A higher HFA-PEFF score was associated with a higher risk of all-cause death (HR 1.390, 95%CI 1.240, 1.558, *P *< 0.001).

**Conclusion:**

The HFA-PEFF score might be applied in HFpEF diagnosis and all-cause death prediction. More studies are required for finding validation.

## Introduction

The prognosis of heart failure patients is poor, stratified according to ejection fraction classification (1). Heart failure with preserved ejection fraction (HFpEF) is a common clinical syndrome, influencing half of all heart failure patients globally, with rising prevalence and significant morbidity and mortality (2, 3). Individuals with HFpEF had a 5-year survival rate of 35%–40% after the first hospitalization (4). Although numerous attempts have been made to find an effective targeted therapy for HFpEF, the currently available evidence is inadequate to support specific drug regimens for patients who present with HFpEF (5–8), probably because the fundamental pathophysiology of HFpEF is poorly understood, and a firm diagnosis of HFpEF remains a challenge in real-world practice.

In 2019, the Heart Failure Association (HFA) of the European Society of Cardiology (ESC) proposed the Heart Failure Association Pre-test Assessment, Echocardiography & Natriuretic Peptide, Functional Testing, Final Etiology (HFA-PEFF) algorithm to diagnose HFpEF (9), where the HFA-PEFF score incorporates three domains, functional, morphological, and biomarker, to estimate the likelihood (low, intermediate, or high) of suffering from HFpEF (10). Besides, the strategies for improving outcomes in patients with HFpEF are not well-defined. Better definitions of the population at higher clinical risk may be helpful in adjudicating the intensity of follow-up and optimizing therapies (11). Many clinical, biochemical, and echocardiographic derangements have been linked to worse outcomes, and the HFA-PEFF, which considers several easily available variables, has been shown by the existing studies to be helpful in both diagnosis and prognostic prediction of HFpEF (12–17). At present, Li et al. (4) has comprehensively investigated the diagnostic role of the HFA-PEFF score in HFpEF via a meta-analysis, whereas the prognostic value of the HFA-PEFF score for HFpEF is still unclear.

The objective of this systematic review and meta-analysis was to assess the diagnostic and prognostic performances of the HFA-PEFF score for HFpEF in a comprehensive manner, so as to provide a comprehensive understanding of the HFA-PEFF score and promote the clinical risk management of HFA-PEFF.

## Methods

### Search strategy

The following four English databases were comprehensively searched by two independent investigators (XM Li and YY Liang) from the inception to June 12, 2023: PubMed, Embase, Cochrane Library, and Web of Science. The English search term was HFA PEFF. Primary screening was carried out by reading titles and abstracts of the retrieved studies with the help of Endnote X9 (Clarivate, Philadelphia, PA, USA). Subsequently, full texts were read to select eligible studies. Discussion was needed when differences arose regarding search results. This systematic review and meta-analysis was performed according to the Preferred Reporting Items for Systematic Reviews and Meta-analysis (PRISMA).

### Eligibility criteria

Inclusion criteria included: (1) studies on individuals with suspected HFpEF (for diagnosis analysis) or diagnosed with HFpEF (for prognosis analysis); (2) studies reporting the HFA-PEFF score; (3) studies providing relevant data to calculate sensitivity (SEN), specificity (SPE), positive likelihood ratio (PLR), negative likelihood ratio (NLR), diagnostic odds ratio (DOR), area under the curve (AUC), and prognostic HR values; (4) studies using the “Rule-out” or “Rule-in” approach for diagnosis analysis or studies on cardiovascular events (CVEs, including cardiovascular death, hospitalization for HF decompensation, nonfatal myocardial infarction (MI), unstable angina pectoris, coronary revascularization for a new diagnosis of angina or in-stent restenosis after percutaneous coronary intervention, and nonfatal ischemic stroke) and all-cause death for prognosis analysis; (5) English studies.

Exclusion criteria included: (1) animal experiments; (2) studies involving partial HFpEF patients for prognosis analysis; (3) studies with unextractable data; (4) meta-analyses, reviews, abstracts, and errata.

### Data extraction and quality assessment

Two investigators (XM Li and YY Liang) independently extracted data from the included studies, including the first author, year of publication, study period, study design, sample size, sex (male/female), age (years), body mass index (BMI, kg/m^2^), comorbidities, medications, HFpEF diagnosis, HFA-PEFF assessment, follow-up time (months), and endpoints. A third author (XZ Lin) would settle relevant disagreements. The Quality Assessment of Diagnostic Accuracy Studies (QUADAS-2) tool was adopted to assess the quality of diagnostic accuracy studies, based on the risk of bias and clinical applicability (18). The risk of bias contained patient selection, index test, reference standard, and flow and timing. Clinical applicability consisted of patient selection, index test, and reference standard. Each item was graded as high (risk), low (risk), or unclear (risk).

### Statistical analysis

Statistical analysis was performed using Meta-disc 1.4 (Clinical Biostatistics, Ramony Cajal Hospital, Madrid, Spain), Stata 15.1 (Stata Corporation, College Station, Texas, USA), and Revman 5.4 (The Nordic Cochrane Centre, The Cochrane Collaboration, Copenhagen, Denmark). Results were obtained via direct extraction or indirect calculation. Meta-disc 1.4 was applied to evaluate whether there was a threshold effect. When the Spearman correlation coefficient between the logarithm of sensitivity and the logarithm of 1-specificity showed a strong positive correlation, it indicated the existence of a threshold effect. To assess the diagnostic and prognostic value of the HFA-PEFF score, the SEN, SPE, PLR, NLR, and DOR as well as 95% confidence intervals (CIs) for clinical outcomes were reported. Summary receiver operating characteristic (SROC) curves were generated, and the AUC was calculated with 95%CIs. Besides, for CVEs and all-cause death, the hazard ratio (HR) values were calculated using Stata 15.1 with the HFA-PEFF as a continuous variable. Revman 5.4 was used to create a quality assessment chart for the included studies. Differences were significant when *P* values were less than 0.05.

## Results

### Study characteristics

**Figure 1:**
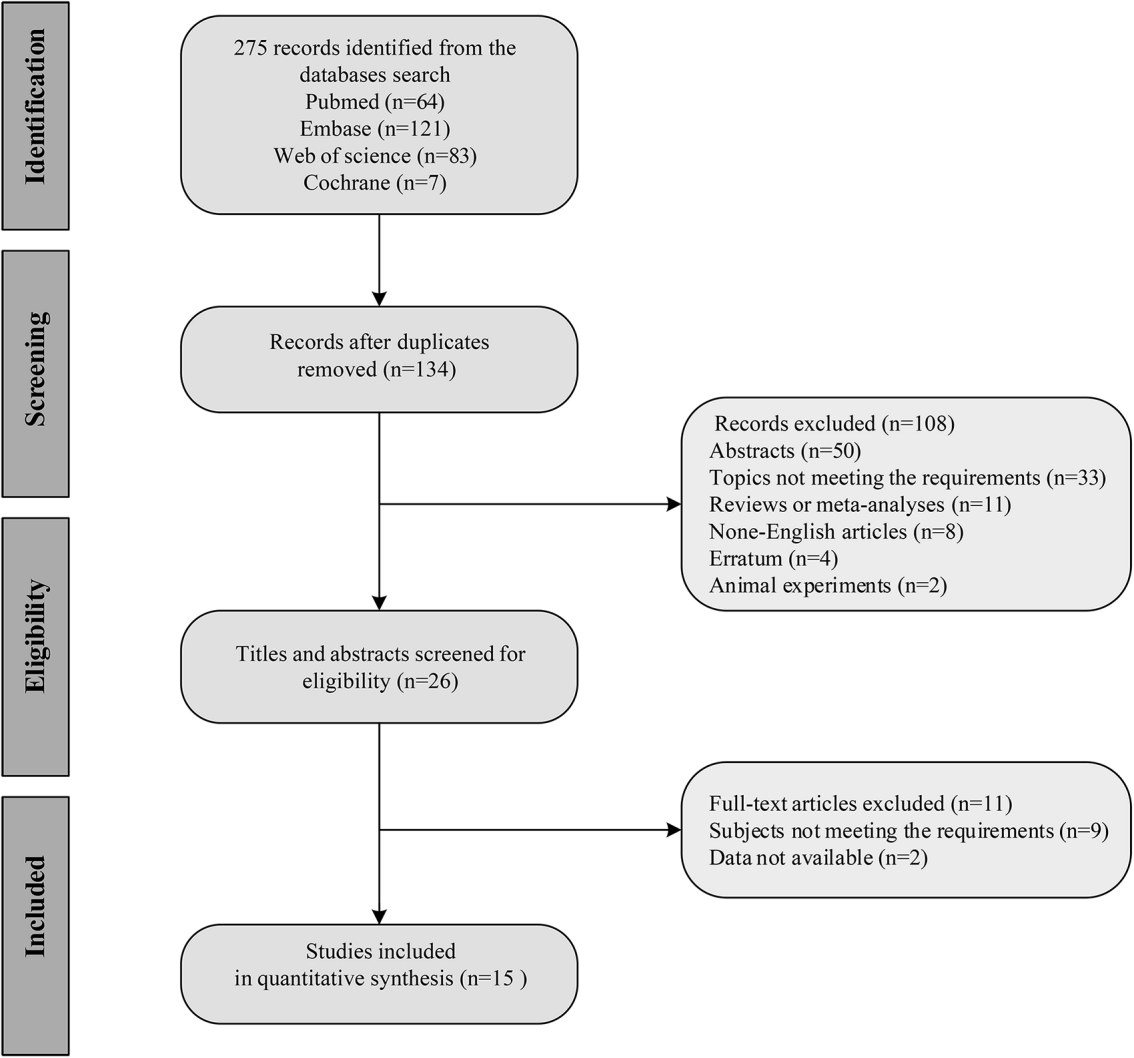
Flow diagram of study screening.

A total of 275 studies were retrieved from the four databases. After excluding duplicates, and based on the eligibility criteria, 15 studies (10, 12–17, 19–26) involving 6,420 subjects were included for this systematic review and meta-analysis in the end, with 5 studies from Japan, 2 from China, 1 from Germany, 1 from Italy, 1 from Poland, 1 from the Netherlands, 1 from Korea, 1 from USA, and 2 from multiple countries. The flow chart of study selection is demonstrated in Figure 1. The year of publication ranged from 2020 to 2023. Seven studies had prospective designs, and eight studies had retrospective designs. Nine studies were involved in diagnosis analysis, and seven were included for prognosis analysis. Table 1 exhibits the detailed characteristics of the included studies. With the QUADAS-2 for the quality assessment of the included studies, as regards the risk of bias and clinical applicability, most studies exhibited low risks, followed by unclear risks (Table 2).

**Table 1:** Characteristics of the included studies.

Author	Year	Study period	Design	Sample size	Sex (M/F)	Age, years	BMI, kg/m^2^	Comorbidities	Medications	HFpEF diagnosis	HFA-PEFF	Follow-up time, months	Endpoints
Choi	2023	2000–2019	Retrospective	404	220/184	65.2 ± 11.4	25.1 ± 3.9	Hypertension 250, diabetes 184, hyperlipidemia 152, CKD 32, AF 44, previous MI 29, previous cerebrovascular accident 20, COPD 11	Beta-blocker 155, ACEI or ARB 185, loop diuretics 62, aldosterone antagonist 41, statin 186, CCB 122, nitrate 60	Patients with HF symptoms, preserved LVEF ≥ 50%, LVEDP ≥ 16 mm Hg	0–1 56, 2–4 226, 5–6 122	120	Diagnosis
Mu	2023	2019–2021	Prospective	128	59/69	65 ± 12	25.8 ± 3.9	Hypertensive 106, CHD 82, AF 24, DM 33	–	Patients with normal ejection fraction and symptoms or signs of heart failure, invasive PCWP ≥15 mm Hg	0–1 27, 2–4 70, 5–6 31	18	Diagnosis
Amanai	2022	2019–2021	Retrospective	187	69/118	68 ± 12	23.7 ± 4.7	Coronary disease 17, DM 32, hypertension 136, AF 41	ACEI/ARB 68, beta-blocker 40, loop diuretics 43	Typical clinical symptoms (dyspnea and fatigue), normal LVEF (>50%), and objective evidence of elevated left heart filling pressures at rest and/or with exercise (at least one of the following: the ASE/EACVI-recommended echocardiographic diastolic dysfunction; E/e′ during exercise >15; or invasively-measured PCWP at rest >15 mmHg and/or with supine ergometry exercise ≥25 mmHg)	0–1 9, 2–4 92, 5–6 63	–	Diagnosis
Egashira	2022	2007–2013	Prospective	502	274/228	71.6 ± 9.5	24.1 ± 3.6	DM 156, hypertension 392, dyslipidemia 391, IHD 266, AF 142	Diuretics 122, ACEI or ARB 313, CCB 290, beta-blocker 224, statin 333	(1) symptoms or signs of HF; (2) normal or mildly reduced LVEF (LVEF > 50% and LV end-diastolic volume index <97 ml/m^2^); (3) evidence of abnormal LV relaxation, filling, diastolic distensibility, and diastolic stiffness	2–4 311, 5–6 191	38.6	CVE
Przewlocka-Kosmala	2022	2012–2015	Retrospective	201	53/148	64.2 ± 8.3	29.6 ± 4.1	–	–	(1) signs and symptoms of HF (dyspnea, fatigue, and exercise intolerance) consistent with New York Heart Association (NYHA) functional class II or III with reduced exercise capacity (<100% of age- and sex predicted normal ranges for peak oxygen consumption); (2) preserved LVEF (≥50%); (3) evidence of diastolic dysfunction	0–4 85, 5–6 116	48 (24–60)	CVE
Reddy	2022	2016–2020	Retrospective	736	293/443	67 ± 13	31.3 ± 7.2	Hypertensive 574, diabetes 133, AF 226	–	Elevated PCWP at rest (≥15 mm Hg) or during exercise (≥25 mm Hg) at cardiac catheterization	0–1 97, 2–6 388	–	Diagnosis
Tomasoni	2022	2011–2021	Retrospective	304	197/107	77 (69–82)	25 (23–29)	Hypertension 186, dyslipidaemia 112, diabetes 54, CAD 47, COPD 24, AF 131	ASA 91, ACEI/ARB 134, beta-blocker 155, MRAs 113, direct oral anticoagulants 72, VKA 48, furosemide 202	ESC 2021	0–1 2, 2–4 71, 5–6 231	19 (8–40)	All-cause death
Nikorowitsch	2021	2016–2019	Prospective	407	187/220	66.0 (59.0–71.5)	27.8 (24.9–31.7)	Hypertension 315, diabetes 66, CAD 79, AF 67, PAD 37	Aldosterone antagonists 9, loop diuretics 29, beta-blocker 123, ACEi/ARBs 186	ESC 2016	0–1 93, 2–4 234, 5–6 31	–	Diagnosis
Parcha	2021	2006–2013	Prospective	951	444/490	–	–	AF 233, COPD 107, diabetes 371, dyslipidaemia 678, hypertension 774, PAD 90	ACEI/ARBs 539, beta-blockers 556, CCB 288, diuretics 623, statin 543	TOPCAT trial: the presence of at least one symptom at the time of screening and one sign in the preceding 12 months, LVEF ≥ 45% obtained within six months prior, either having ≥1 heart failure hospitalization in the prior 12 months or BNP ≥ 100 pg/ml or N-terminal pro-BNP ≥ 360 pg/ml in the 60 days prior to enrollment. RELAX trial: LVEF ≥ 50% measured in the last 12 months with objective evidence of heart failure, had elevated NT-proBNP ≥400 pg/ml or BNP ≥ 200 pg/ml or elevated invasively measured filling pressures [pulmonary capillary wedge pressure (>20 mm Hg on rest or >25 mm Hg on exertion)], alongside peak oxygen consumption ≤60% of the age and sex-adjusted normative values while achieving an exercise respiratory exchange ratio ≥1.0	0–1 41, 2–4 484, 5–6 409	–	Diagnosis, CVE
Seo	2021	2017–2019	Retrospective	286	130/156	81.5 ± 5.1	23.0 ± 3.5	Hypertension 174, diabetes 51, AF 31	–	A history of hospitalization for HF, currently undergoing HF treatment, with a LVEF of ≥50% at the time of registration in whom there were no underlying diseases of HF	0–4 178, 5–6 108	–	Diagnosis
Sotomi	2021	2016–2019	Prospective	871	389/482	82.0 (76.5–87.0)	23.7 (20.8–26.8)	Hypertension 736, dyslipidaemia 356, DM 287, AF 440, CAD 150, MI 65, PAD 48, CKD 341, liver dysfunction 56, malignant tumour 100, hypertrophic cardiomyopathy 32, stroke 121	ACEI/ARBs 469, beta-blockers 473, CCB 414, vasodilators 78, diuretics 698, MRA 327, digitalis 30, antiarrhythmic drug 71, anticoagulants 506, antiplatelets 261, sodium-glucose transport protein 2 inhibitor 42, statin 289	(1) clinical symptoms and signs according to the Framingham Heart Study criteria; and (2) serum NT-proBNP level of ≥400 pg/ml or brain natriuretic peptide level of ≥100 pg/ml	0–1 20, 2–4 317, 5–6 487	13.3 ± 11.6	All-cause death
Sun	2021	2015–2018	Retrospective	358	150/208	70.21 ± 8.64	26.87 ± 3.96	AF 191, hypertension 289, diabetes 175, prior MI 84, angina 53	Loop diuretics 228, beta-blockers 263, ACEI 109, ARB 115, spironolactone 182, CCB 138, digoxin 31, stain 221, antiplatelet drug 159	ESC 2016	0–2 63, 3–4 156, 5–6 139	26.9 ± 11.1	All-cause death
Tada	2021	2012–2020	Prospective	372	173/199	71.7 ± 13.3	23.0 ± 4.6	Hypertension 220, DM 109, hyperlipidemia 131, stroke 45, COPD 27, AF 122, HF 64	ACEI/ARBs 157, beta-blockers 140, CCB 150, MRAs 74, loop diuretics 165, statin 94	Hospitalization with a diagnosis of acute decompensated HF according to the Framingham criteria by at least two experienced cardiologists, with LVEF ≥ 50% by the modified Simpson method or LV fractional shortening ≥25% by echocardiography	0–1 19, 2–4 198, 5–6 155	–	Diagnosis
Verbrugge	2021	2010–2015	Retrospective	443	177/266	78 ± 12	34.6 ± 10.1	Diabetes 274, CAD 229, AF 262	RAS blocker 223, beta-blocker 312, MRA 28, loop diuretic 289	At the time of first HF hospitalization, have an echocardiography result within 1 year of admission that demonstrated a LVEF ≥ 50%, received intravenous loop diuretics within 24 h of admission and for a duration of ≥48 h	2–4 49, 5–6 364	28 (8–59)	All-cause death
Aizpurua	2020	2015–2018	Prospective	270	90/180	75.0 ± 8.5	30.4 ± 5.9	Hypertension 227, hyperlipemia 151, DM 92, AF 140, valvular heart disease 108, CAD 63, stroke 37, COPD 45, sleep apnoea 101	ACEI/ARB 130, beta-blocker 112, aldosterone antagonist 25, loop diuretic 85, thiazide 39, statin 83, platelet aggregation inhibitor 40, oral anticoagulant 92	The presence of HF symptoms with a LVEF ≥ 50% at time of inclusion, combined with significant cardiac structural [increased left atrial volume index (LAVI > 34 ml/m^2^) or left ventricular mass index (LVMI ≥ 115 g/m^2^ for men or ≥95 g/m^2^ for women)] or functional abnormalities (mean E/e′ ≥ 13 and/or mean e′ septal and lateral wall <9 cm/s) with additional increased levels of NT-proBNP; or not have increased NT-proBNP but have a previous HF hospitalization or clinical signs of congestion with positive response to diuretic therapy	0–1 11, 2–4 98, 5–6 161	–	Diagnosis

ACEI, angiotensin-converting enzyme inhibitors; AF, atrial fibrillation; ARB, angiotensin-receptor blockers; ASA, acetylsalicylic acid; ASE, American Society of Echocardiography; BMI, body mass index; BNP, B-type natriuretic peptide; CAD, coronary artery disease; CCB, calcium channel blocker; CHD, coronary heart disease; CKD, chronic kidney disease; COPD, chronic obstructive pulmonary disease; DM, diabetes mellitus; EACVI, European Association of Cardiovascular Imaging; HF, heart failure; IHD, ischemic heart disease; LVEDP, left ventricular end- diastolic pressure; LVEF, left ventricular ejection fraction; PAD, peripheral artery disease; PCWP, pulmonary capillary wedge pressure; MI, myocardial infarction; MRA, mineralocorticoid receptor antagonist; NT-proBNP, N-terminal pro-B-type NP; RAS, renin–angiotensin system; VKA, vitamin K antagonist.

**Table 2:** Quality assessment of the included studies by the QUADAS-2.

Study	Risk of bias				Applicability		
	Patient selection	Index test	Reference standard	Flow and timing	Patient selection	Index test	Reference standard
Choi et al. (12)	L	U	L	L	L	U	L
Mu et al. (14)	L	U	L	L	L	U	L
Amanai et al. (19)	L	U	H	H	L	L	H
Egashira et al. (13)	L	L	L	L	L	L	L
Przewlocka-Kosmala et al. (15)	L	L	L	L	L	U	L
Reddy et al. (16)	L	U	U	U	L	U	L
Tomasoni et al. (17)	L	L	L	L	L	L	L
Nikorowitsch et al. (20)	L	U	L	U	L	L	L
Parcha et al. (21)	U	H	U	H	H	H	L
Seo et al. (22)	U	U	L	L	L	H	L
Sotomi et al. (23)	L	L	L	U	L	L	L
Sun et al. (24)	L	L	L	L	L	L	L
Tada et al. (25)	U	L	L	U	L	L	L
Verbrugge et al. (26)	L	L	L	L	L	U	L
Aizpurua et al. (10)	L	U	L	L	L	U	L

QUADAS-2, quality assessment of diagnostic accuracy studies; L, low risk; H, high risk; U, unclear risk.

### Diagnostic performance of the HFA-PEFF score

#### “Rule-out” approach

**Figure 2:**
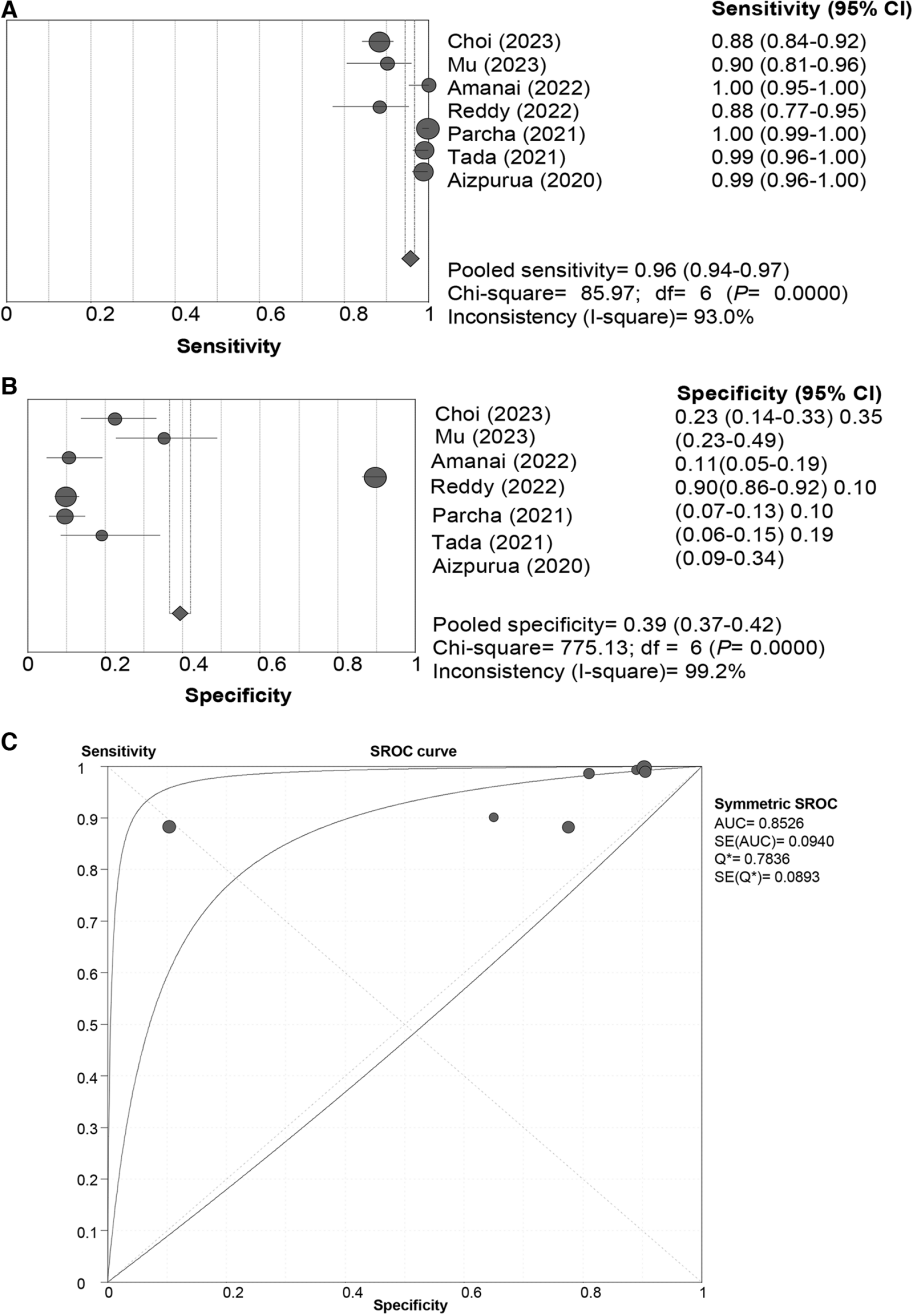
Sensitivity (**A**), specificity (**B**) and SROC curve (**C**) of the HFA-PEFF score for hFpEF diagnosis using the “rule-out” approach. SROC, summary receiver operating characteristic; HFA-PEFF, heart failure association pre-test assessment, echocardiography & natriuretic peptide, functional testing, final etiology; HFpEF, heart failure with preserved ejection fraction; AUC, area under the curve; CI, confidence interval.

In the pooled analysis of the “Rule-out” approach, the SROC curve of the HFA-PEFF showed a “shoulder-arm” distribution, and further, the Spearman correlation coefficient for the HFA-PEFF was 0.786 (*P *= 0.036), which indicated the existence of a threshold effect. The pooled SEN was 0.96 (95%CI: 0.94, 0.97), the pooled SPE was 0.39 (95%CI: 0.37, 0.42), the pooled PLR was 1.47 (95%CI: 1.21, 1.77), the pooled NLR was 0.14 (95%CI: 0.06, 0.33), the pooled DOR was 12.90 (95%CI: 3.78, 44.02), and the pooled AUC was 0.85 (95%CI: 0.67, 1.00) (Table 3; Figure 2).

**Table 3:** Diagnostic and prognostic performances of the HFA-PEFF score for hFpEF.

Indicators	SEN	SPE	PLR	NLR	DOR	AUC	Threshold effect
Diagnostic performance							
Rule out	0.96 (0.94, 0.97)	0.39 (0.37, 0.42)	1.47 (1.21, 1.77)	0.14 (0.06, 0.33)	12.90 (3.78, 44.02)	0.85 (0.67, 1.00)	*r* = 0.786, *P *= 0.036
Rule in	0.59 (0.56, 0.61)	0.86 (0.84, 0.88)	4.93 (3.69, 6.60)	0.46 (0.35, 0.60)	11.38 (8.71, 14.85)	0.83 (0.79, 0.87)	*r* = 0.881, *P *= 0.004
Prognostic performance							
CVE	0.63 (0.58, 0.67)	0.53 (0.49, 0.58)	1.50 (1.07, 2.10)	0.44 (0.17, 1.13)	3.38 (1.15, 9.96)	0.65 (0.40, 0.90)	*r* = 0.5, *P *= 0.667
All-cause death	0.85 (0.81, 0.88)	0.48 (0.44, 0.52)	1.34 (1.12, 1.59)	0.47 (0.29, 0.76)	2.96 (1.73, 5.06)	0.65 (0.47, 0.83)	*r* = 0.5, *P *= 0.667

SEN, sensitivity; SPE, specificity; PLR, positive likelihood ratio; NLR, negative likelihood ratio; DOR, diagnostic odds ratio; AUC, area under the curve.

#### “Rule-in” approach

**Figure 3:**
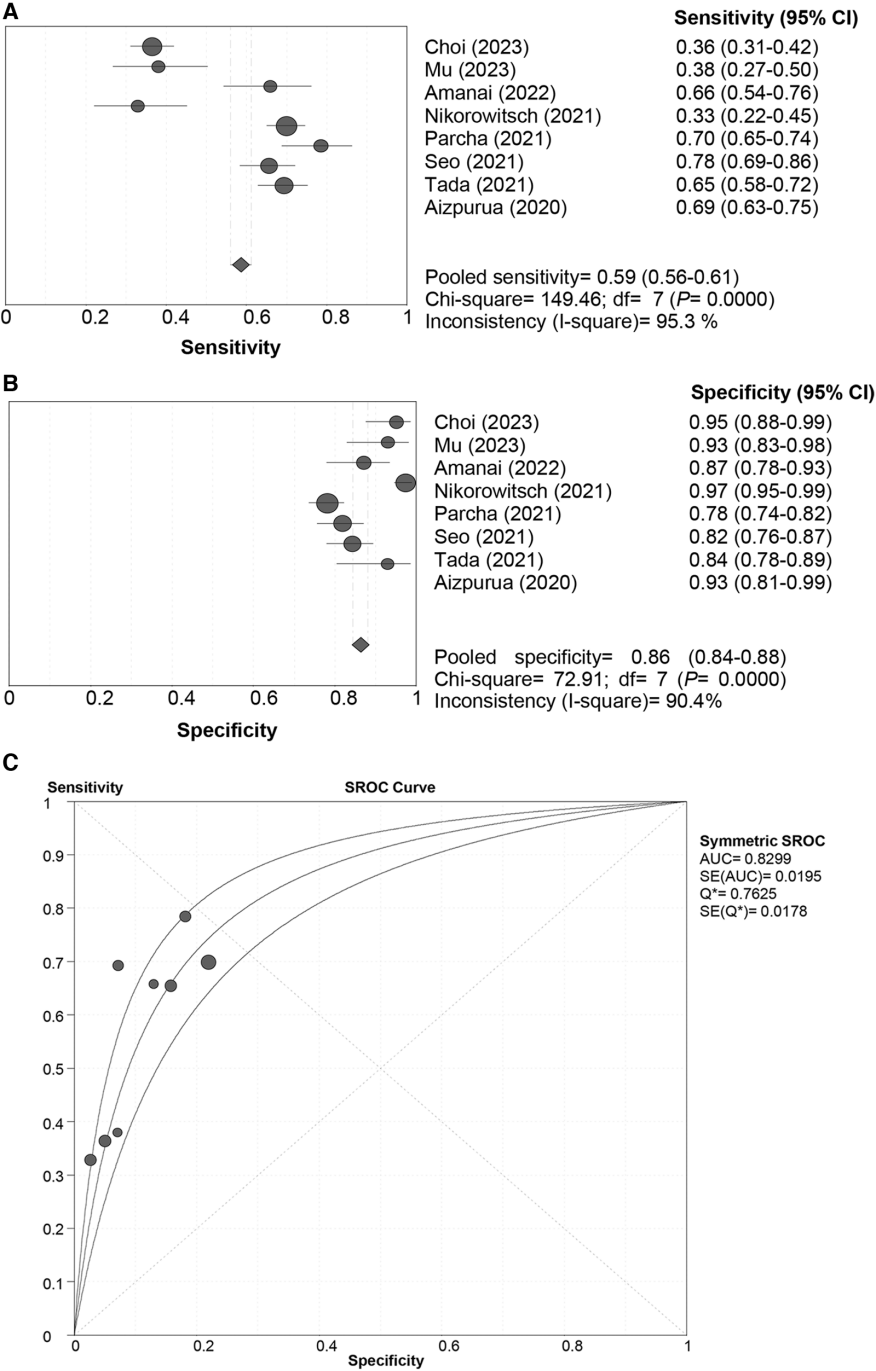
Sensitivity (**A**), specificity (**B**) and SROC curve (**C**) of the HFA-PEFF score for hFpEF diagnosis using the “rule-in” approach. SROC, summary receiver operating characteristic; HFA-PEFF, heart failure association pre-test assessment, echocardiography & natriuretic peptide, functional testing, final etiology; HFpEF, heart failure with preserved ejection fraction; AUC, area under the curve; CI, confidence interval.

In the pooled analysis of the “Rule-in” approach, a “shoulder-arm” distribution was illustrated by the SROC curve of the HFA-PEFF. The Spearman correlation coefficient for the HFA-PEFF was 0.881 (*P *= 0.004), suggesting the existence of a threshold effect. The pooled SEN was 0.59 (95%CI: 0.56, 0.61), the pooled SPE was 0.86 (95%CI: 0.84, 0.88), the pooled PLR was 4.93 (95%CI: 95%CI: 3.69, 6.60), the pooled NLR was 0.46 (95%CI: 0.35, 0.60), the pooled DOR was 11.38 (95%CI: 8.71, 14.85), and the pooled AUC was 0.83 (95%CI: 0.79, 0.87) (Table 3; Figure 3).

### Predictive performance of the HFA-PEFF score

#### CVEs

**Figure 4:**
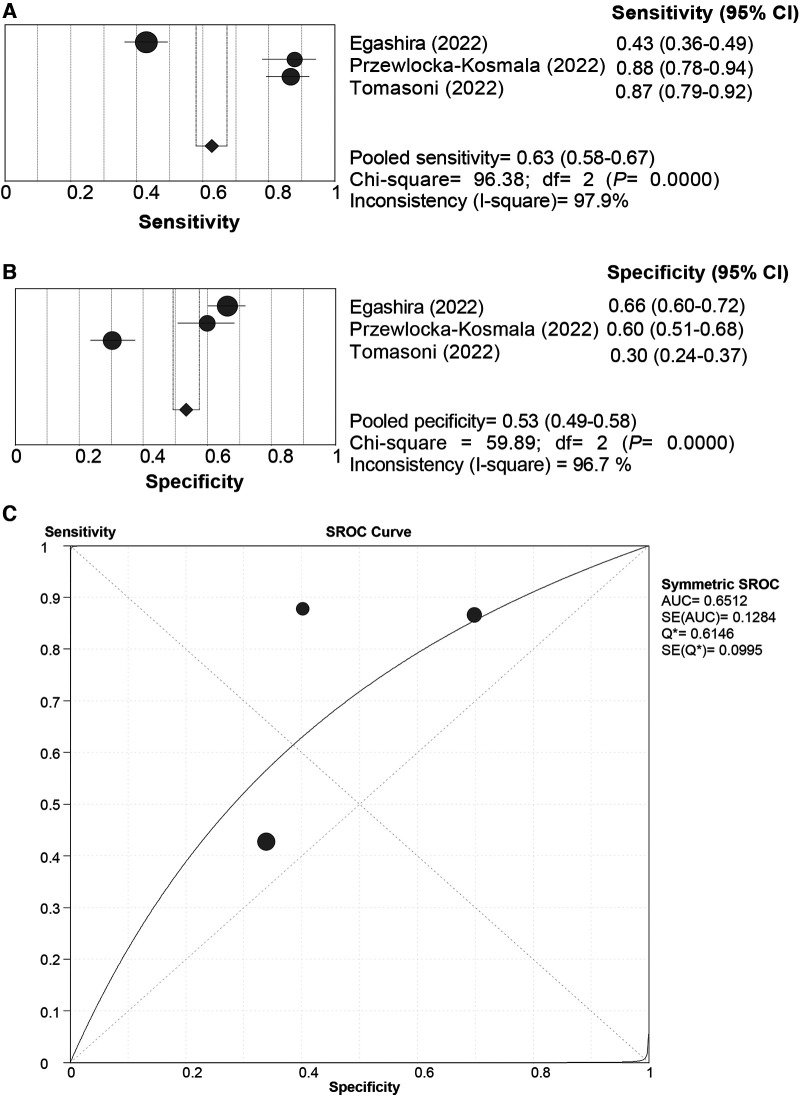
Sensitivity (**A**), specificity (**B**) and SROC curve (**C**) of the HFA-PEFF score for CVE prediction. SROC, summary receiver operating characteristic; HFA-PEFF, heart failure association pre-test assessment, echocardiography & natriuretic peptide, functional testing, final etiology; HFpEF, heart failure with preserved ejection fraction; AUC, area under the curve; CI, confidence interval.

All the included studies used the “Rule-in” approach for CVE prediction. The SROC curve of the HFA-PEFF did not show a “shoulder-arm” distribution, and further, the Spearman correlation coefficient for the HFA-PEFF was 0.5 (*P *= 0.667), which indicated the absence of a threshold effect. The pooled SEN was 0.63 (95%CI: 0.58, 0.67), the pooled SPE was 0.53 (95%CI: 0.49, 0.58), the pooled PLR was 1.50 (95%CI: 1.07, 2.10), the pooled NLR was 0.44 (95%CI: 0.17, 1.13), the pooled DOR was 3.38 (95%CI: 1.15, 9.96), and the pooled AUC was 0.65 (95%CI: 0.40, 0.90) (Table 3; Figure 4). The pooled analysis of 2 eligible studies found no significant association between the HFA-PEFF and the risk of CVEs (HR 1.631, 95%CI 0.984, 2.704, *P *= 0.058).

#### All-cause death

**Figure 5:**
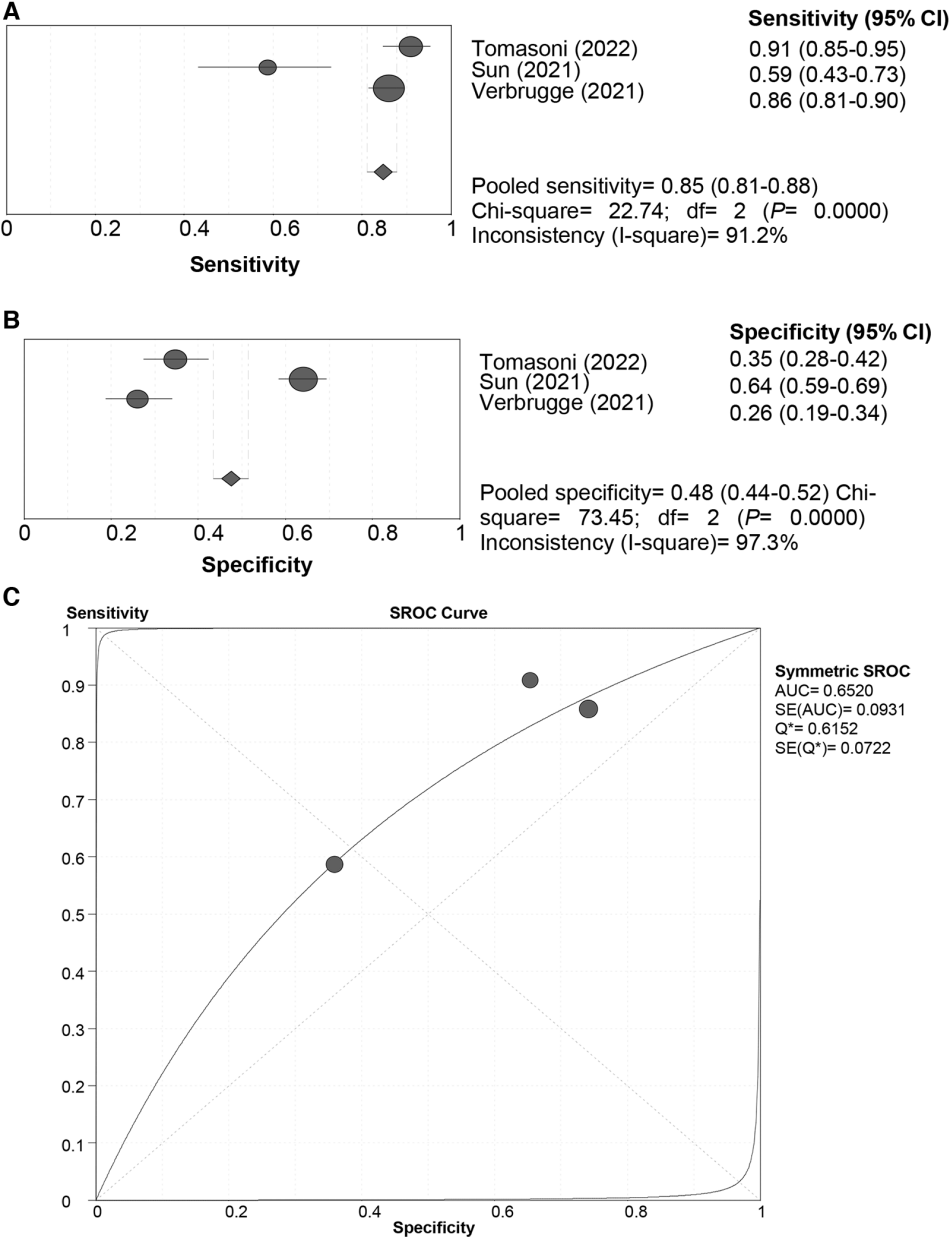
Sensitivity (**A**), specificity (**B**) and SROC curve (**C**) of the HFA-PEFF score for all-cause death prediction. SROC, summary receiver operating characteristic; HFA-PEFF, heart failure association pre-test assessment, echocardiography & natriuretic peptide, functional testing, final etiology; HFpEF, heart failure with preserved ejection fraction; AUC, area under the curve; CI, confidence interval.

All the included studies used the “Rule-in” approach for all-cause death prediction. The SROC curve of the HFA-PEFF did not display a “shoulder-arm” distribution, and further, the Spearman correlation coefficient for the HFA-PEFF was 0.5 (*P *= 0.667), suggesting no threshold effect. The pooled SEN was 0.85 (95%CI: 0.81, 0.88), the pooled SPE was 0.48 (95%CI: 0.44, 0.52), the pooled PLR was 1.34 (95%CI: 1.12, 1.59), the pooled NLR was 0.47 (95%CI: 0.29, 0.76), the pooled DOR was 2.96 (95%CI: 1.73, 5.06), and the pooled AUC was 0.65 (95%CI: 0.47, 0.83) (Table 3; Figure 5). The pooled analysis of 3 qualified studies demonstrated that a higher HFA-PEFF score was associated with a higher risk of all-cause death (HR 1.390, 95%CI 1.240, 1.558, *P *< 0.001).

## Discussion

The current systematic review and meta-analysis comprehensively evaluated the diagnostic and prognostic value of the HFA-PEFF score for HFpEF, and illustrated that with both the “Rule-out” and the “Rule-in” approaches, the HFA-PEFF had a good diagnostic capability for HFpEF based on the pooled AUCs, and the pooled SEN of the “Rule-out” approach was higher than that of the “Rule-in” approach, while the pooled SPE of the “Rule-in” approach was better than that of the “Rule-out” approach; for all-cause death, the HFA-PEFF exhibited a good predictive SEN, which indicated that the HFA-PEFF might be applied in HFpEF diagnosis and all-cause death prediction.

HFpEF diagnosis is usually performed based on three key components. These include symptoms and signs of heart failure (related to pulmonary and systemic congestion), evidence of “preserved ejection fraction”, and the presence of diastolic dysfunction (27). The HFA-PEFF score algorithm was proposed based on a comprehensive diagnostic workup (9, 10). In addition, this score also demonstrated its prognostic significance for individuals with HFA-PEFF in several studies (13, 23, 24). A prior meta-analysis pooled relevant studies to synthetically assess the diagnostic performance of the HFA-PEFF for HFpEF, and it was found that the HFA-PEFF algorithm showed acceptable SPE and SEN for the diagnosis and exclusion of HFpEF (4). This study further conducted an updated comprehensive analysis of HFA-PEFF prognostic value in HFpEF, and the HFA-PEFF was also found to have great performance in HFpEF diagnosis and all-cause death prognostication.

For the diagnosis of HFpEF, the pooled AUC, SEN and SPE of the “Rule-out” approach was 0.85, 0.96 and 0.39, respectively, and the pooled AUC, SEN and SPE of the “Rule-in” approach was 0.83, 0.59 and 0.86, respectively. These suggested that using either the “Rule-out” approach or the “Rule-in” approach, the HFA-PEFF score had a good diagnostic performance; the “Rule-out” approach showed higher SEN, and the “Rule-in” approach had better SPE. To be noted, for patients with intermediate likelihood of HFpEF, exercise induced echocardiography or invasive cardiac hemodynamic measurements would be recommended for the final diagnosis (25). A two-center study has discovered that exercise pulmonary ultrasound exhibits excellent diagnostic value for HFpEF, regardless of the exercise protocol or level of expertise (28). More precise diagnostic modalities are needed, especially in such a serious disease with an incidence close to the incident rate of tuberculosis and other plagues (29–32), and the clinical indications would guide the diagnostic practice in the context of individualized medical management. Future studies are warranted to verify the diagnostic role of the HFA-PEFF score.

However, in a case-control study evaluating outpatient dyspnea of indeterminate cause and HFpEF, the H2FPEF score exhibited better diagnostic performance compared to the HFA-PEFF score (16). A study based on a Japanese patient cohort has found that the H2FPEF score significantly outperforms the HFA-PEFF score in terms of diagnostic accuracy for HFpEF (25). A research investigation into the diagnostic utility of the H2FPEF scoring system and the HFA-PEFF E-level scoring system within the context of HFpEF has revealed that both scores are efficacious in either excluding or establishing a definitive diagnosis of HFpEF (33). These results suggest the need for further research to compare the importance of different scoring systems in diagnosing HFpEF.

With respect to prognosis in HFpEF, the HFA-PEFF presented a pooled SEN of 0.85 in the prediction of all-cause death, although the pooled AUC was 0.65, while for CVEs, the HFA-PEFF did not have a favorable predictive ability. As reported by Seoudy et al. (34), the HFA-PEFF score is associated with all-cause mortality and heart failure rehospitalization in patients with preserved ejection fraction after transcatheter aortic valve implantation. The HFA-PEFF score has three components that each contribute equally to the overall score (9). One component relies upon natriuretic peptide levels, which have been shown to be strongly related to adverse clinical outcomes in HFpEF as well as HFrEF (35, 36). The second component of the score relies on morphological criteria such as left atrial volume index and left ventricular mass. Left atrial volume index in particular has been demonstrated to be among the most powerful echocardiography predictors of future HF events and reflects the risk of mortality as well (37–39). Finally, the third component of the HFA-PEFF score is represented by functional parameters that reflect left ventricular diastolic dysfunction and/or elevated cardiac filling pressures. One of the parameters incorporated is tricuspid valve regurgitation velocity, with high values indicating pulmonary hypertension, which is strongly associated with mortality in HFpEF (40). For every one point increase in the HFA-PEFF score, the risk of all-cause mortality significantly increased by 39%, which showed a quantitative information to facilitate understanding of the association between the HFA-PEFF and the death risk. Corresponding optimized treatments could be provided to high-risk patients with HFpEF to improve their prognosis.

As demonstrated by this study, the HFA-PEFF score may be taken into consideration by clinicians in the diagnosis and all-cause death prognostication of HFpEF, which may help in planning therapeutic methods and improving HFpEF management. Several limitations should be mentioned when interpreting the results. First, there were threshold effects on the diagnostic performance of the HFA-PEFF score for HFpEF using the “Rule-out” and “Rule-in” approaches, which may affect the stability of the results. Second, the availability of a limited number of studies for outcomes such as CVEs may have influenced the reliability of our findings. The small sample size within these studies could have reduced the statistical power to detect significant associations, and the heterogeneity across studies in terms of design, population characteristics, and follow-up duration further complicates the interpretation of the results. Third, an important limitation to consider in the validation of the HFA-PEFF score is the heterogeneity of criteria used by each study to define HFpEF patients. The included studies span a period from 2000 to 2021, during which time diagnostic and classification criteria for HFpEF have evolved. This variability in the definition of HFpEF could introduce bias and affect the comparability of results across studies. Our findings highlight the urgent need for additional studies to evaluate the prognostic value of the HFA-PEFF score in HFpEF, with the aim of providing a more robust evidence base to support its use in clinical practice.

## Conclusion

The HFA-PEFF had a good diagnostic capability for HFpEF using both the “Rule-out” and the “Rule-in” approaches based on the pooled AUCs, and it exhibited a good predictive SEN for all-cause death in patients with HFpEF, suggesting that the HFA-PEFF may be considered in HFpEF diagnosis and all-cause death prediction. More studies are needed for finding validation.

## Data Availability

The original contributions presented in the study are included in the article/Supplementary Material, further inquiries can be directed to the corresponding author.
